# Mechanistic Insights into the Antihypertensive and Cardioprotective Actions of Corosolic Acid: A Narrative Review

**DOI:** 10.3390/molecules31162841

**Published:** 2026-08-14

**Authors:** Fangying Chen, Wan Yin Tew, Ming Thong Ong, Mun Fei Yam

**Affiliations:** 1 Institute for Research in Molecular Medicine (INFORMM), Universiti Sains Malaysia, Gelugor 11800 USM, Pulau Pinang, Malaysia; chenfangyingbicf@163.com; 2 Department of Pharmacology, School of Pharmaceutical Sciences, Universiti Sains Malaysia, Gelugor 11800 USM, Pulau Pinang, Malaysia; wytew0507@gmail.com

**Keywords:** corosolic acid, hypertension, renin–angiotensin system, oxidative stress, vasodilation, metabolic syndrome

## Abstract

Background: The specific mechanisms underlying the antihypertensive actions of corosolic acid (CA) have not been systematically elucidated. Methods: Evidence was categorized according to major signaling and physiological pathways, including the renin–angiotensin system (RAS), oxidative and inflammatory responses, endothelial and smooth muscle regulation (NO/cGMP and H_2_S/K_ATP_), and kinase-mediated signaling (AMPK, NF-κB, JAK/STAT, PKC). Results: Consistent with in vitro and animal studies, CA has been reported to attenuate pro-hypertensive signaling through multiple pathways: (i) downregulating renin–angiotensin system-related cascades; (ii) reduction in reactive oxygen species and inflammatory mediators through activation of AMPK and inhibition of NF-κB and JAK/STAT pathways; (iii) improved vascular tone by enhancing NO/cGMP signaling, partly involving the H_2_S/K_ATP_ pathway, and inhibiting PKC. CA may indirectly contribute to blood pressure reduction by improving glucose and lipid metabolism and adipose tissue inflammation, thereby affecting metabolic risk factors. The existing evidence gaps include: lack of direct studies on voltage-gated, receptor-gated and store-regulated calcium channels; the specific effects on the isoforms of nitric oxide synthase (eNOS/iNOS/nNOS) and PKC are not known; limited human data on the antihypertensive efficacy, dose effect and safety of CA in humans are available. Conclusions: CA may have multi-targeted blood-pressure-lowering potential. Its clinical efficacy and safety require further confirmation.

## 1. Introduction

Hypertension is one of the most common non-communicable diseases worldwide and is a major risk factor for the morbidity and mortality of cardiovascular diseases [1,2,3]. Although there are various antihypertensive drugs available, such as angiotensin-converting enzyme inhibitors, beta-blockers, and calcium channel blockers, the long-term control of blood pressure remains unsatisfactory in many populations [4,5,6]. Therefore, in recent years, the search for complementary or adjuvant therapies from natural products with multi-target potential and good safety has attracted increasing attention [7,8,9].

Corosolic acid (CA) is a pentacyclic triterpenoid compound that is not limited to *Lagerstroemia speciosa* (banaba) but has been reported in a wider range of medicinal and edible plants. In addition to *Lagerstroemia speciosa*, commonly cited CA-containing plants include hawthorn (*Crataegus pinnatifida*), guava (*Psidium guajava*), loquat (*Eriobotrya japonica*), cat’s whiskers (*Orthosiphon stamineus*), apricot (*Prunus armeniaca*), chokecherry (*Prunus padus*), eucalyptus (*Eucalyptus globulus*), Chinese kiwi (*Actinidia chinensis*), terminalia (*Terminalia chebula*), solanum (*Solanum torvum*), osmanthus (*Osmanthus fragrans*), and rosemary (*Salvia rosmarinus*) [10,11,12]. Early studies focused primarily on the antidiabetic and anti-inflammatory properties of CA [10,13,14,15]. Recent experimental evidence suggests that CA may also exert cardioprotective and hypotensive effects through a variety of molecular mechanisms [16,17,18]. These mechanisms may include regulating downstream RAS-related signaling pathways [19,20], alleviating oxidative stress and inflammation [21,22] and enhancing the bioavailability of endothelial nitric oxide (NO) [23]. In addition, studies have shown that CA can also regulate several key signaling pathways, such as AMP-activated protein kinase (AMPK) [24,25], peroxisome proliferator-activated receptor γ (PPAR-γ) [16], nuclear factor κB (NF-κB) [26,27], Janus kinase/signal transducer and activator of transcription (JAK/STAT) [28], and protein kinase C (PKC) [29]. These pathways are all involved in maintaining vascular homeostasis. Therefore, this review focuses on CA as a compound itself, rather than limiting the evidence to CA derived from *Lagerstroemia speciosa*.

Although these findings suggest that CA may be a multi-targeted candidate compound for lowering blood pressure, the current data are scattered across different experimental systems. Most studies focus on glucose metabolism [13,30], inflammation [31], or oxidative stress [11,32], and only indirectly involve blood pressure regulation. Therefore, this review integrates available evidence into a unified mechanistic framework linking vascular, endothelial, and cardiometabolic pathways involved in blood pressure regulation. Unlike previous reviews that emphasize primarily metabolic or anti-inflammatory effects of CA, this work specifically focuses on vascular signaling mechanisms underlying its antihypertensive actions. By synthesizing findings from in vitro and in vivo studies, this review provides an updated conceptual framework for understanding the role of CA in vascular regulation and identifies key gaps for future preclinical and clinical research.

## 2. Material and Methods

### 2.1. Literature Search Strategy

A structured literature search was performed with the purpose of finding those papers which evaluated the pharmacologic effect of CA against hypertension and cardiac activity. Electronic databases (PubMed, Scopus, Web of Science, and Google Scholar) were searched for papers that have been published up to August 2025. To avoid restricting the search to a single botanical source, the search strategy included both the compound name and representative CA-containing plant species. The search terms and Boolean logic were as follows: (“corosolic acid” OR “2α,3β-dihydroxyurs-12-en-28-oic acid” OR “*Lagerstroemia speciosa*” OR “banaba” OR “*Crataegus pinnatifida*” OR “*Psidium guajava*” OR “*Eriobotrya japonica*” OR “*Orthosiphon stamineus*” OR “*Prunus armeniaca*” OR “*Prunus padus*” OR “*Eucalyptus globulus*” OR “*Actinidia chinensis*” OR “*Terminalia chebula*” OR “*Solanum torvum*” OR “*Osmanthus fragrans*” OR “*Salvia rosmarinus*”) AND (“hypertension” OR “blood pressure” OR “vascular function” OR “endothelial function” OR “renin-angiotensin system” OR “oxidative stress” OR “nitric oxide” OR “inflammation” OR “protein kinase” OR “cardioprotection”). Reference lists of selected articles and recent reviews were also screened in order not to miss relevant papers. The search strategy was prospectively registered in PROSPERO database, ID: CRD420261329130.

### 2.2. Inclusion and Exclusion Criteria

Studies were selected based on relevance to cardiovascular outcomes, as well as review articles which discussed the possible effects of CA or plant extracts containing CA in lowering blood pressure (BP), prevent heart failure, or relax blood vessels in vitro, in vivo, or ex vivo. Studies of the association between CA and certain cellular structures, its role as part of signal transduction pathways, or the mechanism of action related to oxidation stress, inflammation, regulation of metabolism was included. But studies that focus solely on non-cardiovascular related drug action (e.g., anti-cancer, anti-bacterial, etc.), unpublished reports or non-English-language articles did not qualify for inclusion.

### 2.3. Data Extraction and Synthesis

Inclusion criteria: The data of relevant study were extracted including type of study design, dose or concentration of CA, targeted pathway and relevant biomarker/enzymes involved, and the main experimental findings. We then categorized these results according to their biological mechanism of action, including: the RAS modulation, antioxidant activity and anti-inflammatory effects, control of vasodilatory pathways and attenuation of risk factors of metabolism and cardiovasular disease. Due to high heterogeneity among reports, we performed a narrative review of the findings to highlight converging results at the cellular and/or molecular level, rather than performing a meta-analysis due to heterogeneity.

### 2.4. Quality Considerations

Given that most included studies were preclinical trials, formal risk-of-bias assessment was not conducted due to narrative nature. However, emphasis was placed on reproducibility of findings across independent laboratories, clarity of experimental design, and mechanistic consistency among different model systems. All exceptions and limitations of the investigated variables were mentioned in discussion part, which will help future researchers in their study.

## 3. Outline of CA: Structure and Properties

### 3.1. Chemical Identity and Nomenclature

CA is a pentacyclic triterpenoid compound with the molecular formula C_30_H_48_O_4_ and a molecular weight of 472.7 g/mol. Its IUPAC systematic name is: (1S, 2R, 4aS, 6aR, 6aS, 6bR, 8aR, 10R, 11R, 12aR, 14bS)-10,11-dihydroxy-1,2,6a,6b,9,9,12a-heptamethyl-2,3,4,5,6,6a,7,8,8a,10,11,12,13,14b-tetradecahydro-1H-picene-4a-carboxylic acid (CAS No. 4547-24-4). It belongs to the betulinic acid type triterpenoids and is structurally related to betulin. It has a hydroxyl group at C-2 and a carboxyl group at C-28. CA is lipid-soluble and has poor water solubility. It is usually extracted from *Lagerstroemia speciosa* (banaba), *Crataegus pinnatifida*, *Eriobotrya japonica*, and *Psidium guajava*. These plants have been used in traditional Asian medicine for treating metabolic and cardiovascular diseases for a long time [10,12,33].

Clarifying the chemical properties of CA is helpful for accurately understanding its pharmacokinetic behavior, such as its absorption, distribution, and potential interaction with cell membranes, which is crucial for its biological effects in the vascular system.

### 3.2. Physicochemical and Pharmacological Characteristics

CA belongs to the class of triterpenes derived from the cyclization of squalene. It appears as a white crystalline powder with a melting point around 208–210 °C and is soluble in organic solvents such as methanol, ethanol, and chloroform. Its amphipathic structure allows interaction with biological membranes and enzymatic targets that govern cellular signal transduction [10,12,33]. Pharmacologically, CA has been reported to exhibit a broad spectrum of activities including antidiabetic, antioxidant, anti-inflammatory, antihyperlipidemic, anti-atherosclerotic, antimicrobial, and anticancer effects [26,34,35,36,37,38]. Many of these properties are relevant to cardiovascular protection and may collectively contribute to its blood-pressure-lowering potential.

### 3.3. Therapeutic Applications and Relevance to Hypertension

Early studies on CA focused mainly on its ability to exert hypoglycemic activity by promoting glucose uptake and improving insulin sensitivity, and it was subsequently used as a dietary supplement for the management of type 2 diabetes. Beyond its historical use as an antidiabetic active ingredient, CA-containing medicinal plants offer a broader context for ethnopharmacology and clinical applications. The leaves of *Lagerstroemia speciosa* (banaba) have long been used in traditional Asian medicine and nutritional supplements for the management of diabetes, obesity, and related metabolic diseases. Meanwhile, other CA-containing plants, such as hawthorn (*Crataegus pinnatifida*), loquat (*Eriobotrya japonica*), and guava (*Psidium guajava*), have also been used in traditional medicine for cardiovascular metabolic, inflammatory, or vascular-related diseases [10,13,32,33]. While these historical and dietary records do not constitute direct clinical evidence that purified CA can lower blood pressure, they support the cardiovascular relevance of CA-containing plants and provide a basis for translational research into the role of CA in hypertension-related endothelial dysfunction, oxidative stress, inflammation, and metabolic risk factors. Subsequent research has shown that CA can also alleviate oxidative stress, inflammation [21] and vascular dysfunction, which are key pathophysiological drivers of hypertension and atherosclerosis [37]. By activating AMPK, PPAR-γ and inhibiting NF-κB, CA helps restore endothelial integrity and vascular dilation capacity [16,39]. Although a large number of in vivo and in vitro studies support its potential for cardiac protection, there is currently a lack of systematic review of the molecular pathways related to CA and blood pressure control. Therefore, it is necessary to conduct a comprehensive review of its mechanism of action to clarify the preclinical basis for CA as a potential adjunctive candidate for hypertension management; at the same time, it should be recognized that its clinical applicability has not yet been confirmed due to the lack of human clinical trials and limited pharmacokinetic evidence.

## 4. Essential Mechanisms of CA in Hypertension (Figure 1)

Experimental evidence from in vivo and in vitro studies suggests that CA may exert its blood-pressure-lowering effects through multiple interrelated pathways, which involve vascular tone, oxidative stress, inflammation, and endothelial function. This mechanism may contribute to the overall vasodilatory effect of this compound, producing effects that resemble those observed with traditional renin–angiotensin system inhibitors [19,20]. These mechanisms can be broadly classified into the following categories: regulation of the renin–angiotensin system, reduction in oxidative and inflammatory stress, regulation of vasodilation signaling, and improvement of metabolism and endothelial health.

**Figure 1:**
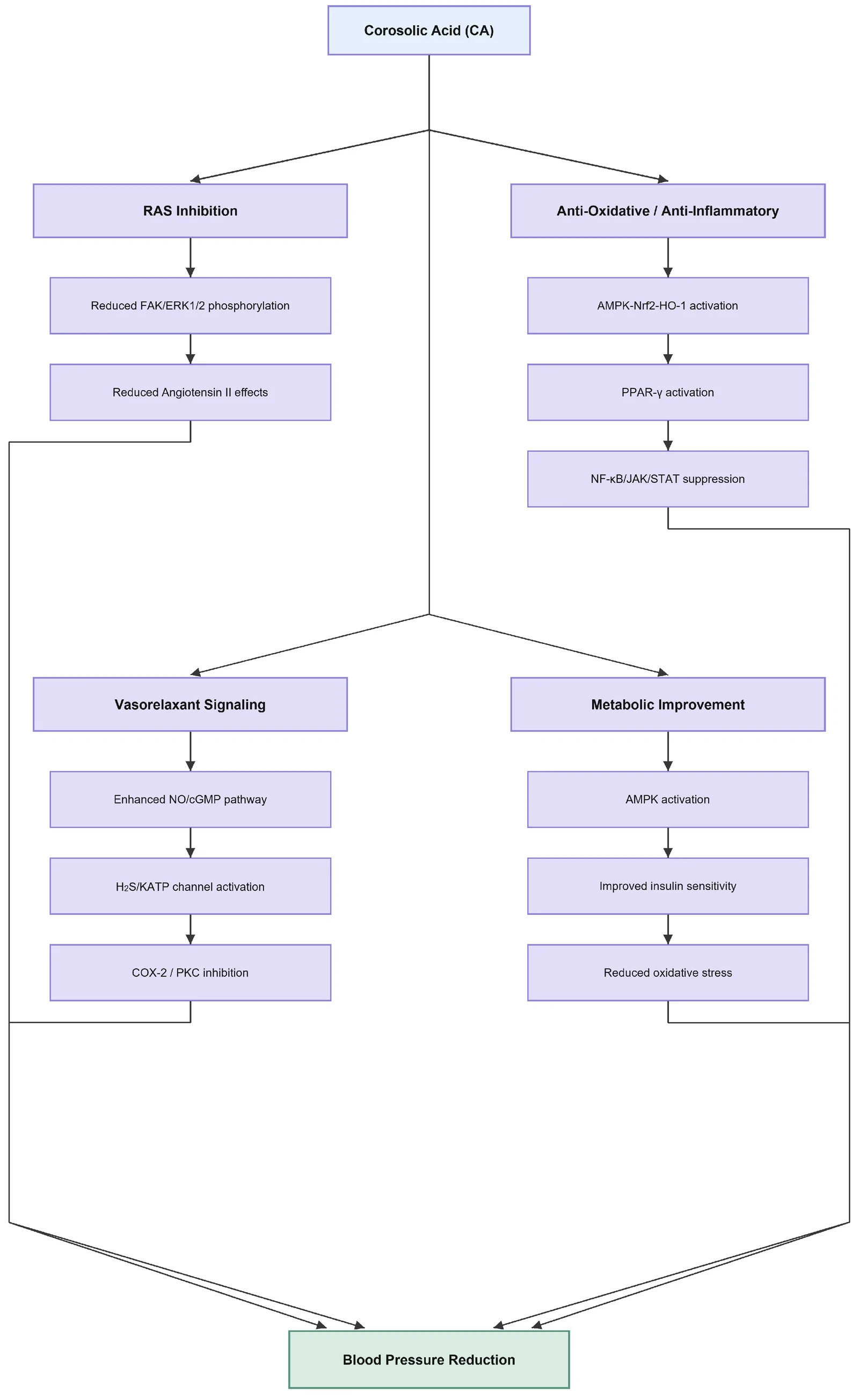
Essential pathways through which corosolic acid (CA) alleviates hypertension. Corosolic acid exerts multi-target antihypertensive actions by modulating interlinked signaling networks. CA may attenuate RAS-related downstream signaling, including focal adhesion kinase/extracellular signal-regulated kinase (FAK/ERK) phosphorylation and vascular remodeling-associated pathways [19,20,40]. A possible interaction between CA and angiotensin II type 1 receptor (AT1R) has also been suggested by in silico evidence, but this requires further biochemical and functional validation [25]. CA activates AMPK leading to Nrf2/HO-1-mediated antioxidant responses [21,41,42], and downregulates NF-κB and JAK/STAT inflammatory cascades [28,43,44]. Enhanced NO/cGMP/PKG [18,23,45] and H_2_S/K_ATP_ signaling pathways [18] promote vasorelaxation, while inhibition of PKC further reduces calcium-dependent vasoconstriction [29,46,47]. CA also improves metabolic profiles through AMPK/PPAR-γ activation and suppression of SREBPs [16,26,27,48,49], collectively restoring endothelial integrity and reducing vascular inflammation.

### 4.1. Modulation of RAS-Related Signaling and Current Evidence Gaps

The classic RAS activation process involves renin mediating the conversion of angiotensinogen to angiotensin I, followed by angiotensin-converting enzyme (ACE) mediating the generation of angiotensin II, which further activates the angiotensin II type 1 receptor (AT1R). This process promotes vasoconstriction, oxidative stress, inflammation, and vascular remodeling. However, current evidence specifically supporting CA for angiotensin II receptor blockade (CA) directly demonstrating its inhibition of renin activity, ACE activity, or AT1R ligand binding remains limited. Therefore, existing evidence is insufficient to support the conclusion that CA can directly block upstream enzymes or receptor components of the RAS. Instead, current research primarily suggests that CA may attenuate downstream RAS-related signaling, particularly FAK/ERK phosphorylation and vascular remodeling-related pathways. Some computer simulations suggest that CA may interact with AT1R, but these findings require further confirmation through biochemical enzyme activity assays, receptor binding assays, and Ang II-induced vasoconstriction functional models. Therefore, the role of CA in RAS regulation should currently be interpreted as indirect modulation of RAS-associated signaling, including FAK/ERK-related pathways [19,20,40], while the possible interaction between CA and AT1R remains based on in silico evidence and requires further biochemical and functional validation [25].

### 4.2. Antioxidative and Anti-Inflammatory Effects

#### 4.2.1. AMPK-Nrf2-HO-1 Pathway

CA enhances the antioxidant defense capacity by activating the adenosine monophosphate-activated protein kinase α (AMPKα)-nuclear factor erythroid 2-related factor 2 (Nrf2)-heme oxygenase-1 (HO-1) signaling axis. This pathway can reduce the generation of reactive oxygen species (ROS) and inhibit the expression of NADPH oxidase (NOX2 and NOX4). The resulting reduction in oxidative stress levels can alleviate endothelial damage and improve vascular reactivity [21,41,42].

#### 4.2.2. PPAR-γ Modulation

CA can activate PPAR-γ. PPAR-γ is a transcription factor that regulates lipid metabolism and vascular inflammation. By inhibiting the phosphorylation of ERK1/2, CA indirectly promotes the activation of PPAR-γ, thereby reducing oxidative stress and enhancing endothelial protection [16].

#### 4.2.3. NF-κB, JAK/STAT, and AMPK Crosstalk

CA exhibits significant anti-inflammatory effects by inhibiting the NF-κB and JAK/STAT pathways and simultaneously activating AMPK. In macrophage and vascular smooth muscle cell models, CA downregulates the expression of pro-inflammatory cytokines (such as interleukin-1β, tumor necrosis factor-α, interleukin-6) and inhibits the activity of key regulatory factors (including inhibitor of κB kinase β (IKKβ) and IL-1 receptor-associated kinase). These effects jointly alleviate endothelial inflammation and vascular remodeling, which are characteristic changes in hypertension [28,43,44].

### 4.3. Regulation of Vasorelaxant Signaling

#### 4.3.1. Activation of Nitric Oxide Synthase (NOS) and the NO/cGMP Pathway

CA has been shown to promote NO production by enhancing the activity of endothelial NOS (eNOS). Subsequently, NO activates soluble guanylyl cyclase (sGC), increasing the intracellular level of cyclic guanosine monophosphate (cGMP) [18,23]. The increase in cGMP levels can activate protein kinase G (PKG), leading to the phosphorylation of contractile proteins (such as heat shock protein 20), thereby causing the relaxation of vascular smooth muscle. Additionally, the S-nitrosylation of caspase-3/8 mediated by NO can reduce cell apoptosis, which is beneficial for maintaining vascular integrity [18,45].

#### 4.3.2. Modulation of H_2_S/K_ATP_ Channel Pathway

CA has been reported to induce vasodilation partly through activation of the hydrogen sulfide (H_2_S)/ATP-sensitive potassium (K_ATP_) pathway. The opening of these channels causes hyperpolarization of vascular smooth muscle cells, reduces calcium ion influx, and leads to vasodilation. This mechanism works in synergy with the NO/cGMP signaling pathway to maintain vascular tone [18].

#### 4.3.3. Cyclooxygenase (COX)-Related Modulation

CA may be involved in COX-related anti-inflammatory effects; however, direct evidence regarding CA’s competitive inhibition of COX-2 remains limited. One study on the structure–activity relationship of pentacyclic triterpenoids included CA in direct COX-1/COX-2 enzyme activity screening; another recent study reported that CA reduces COX-2 expression and showed predictive binding affinity for COX2 in cell models. Nevertheless, these findings do not demonstrate that CA can directly or selectively inhibit vascular COX-2 in hypertension-related models. Therefore, COX-related regulation should currently be understood as a potential anti-inflammatory mechanism, requiring further confirmation using purified CA, direct COX-1/COX-2 enzyme activity assays, prostaglandin/thromboxane assays, and vascular inflammation models [50,51,52].

#### 4.3.4. Inhibition of PKC

In vitro studies have demonstrated that CA inhibited PKC activity in a dose-dependent manner. Since PKC activation enhances vascular contraction by promoting the phosphorylation of contractile proteins and regulating calcium channels, CA may provide an additional vasodilatory mechanism through inhibition of this pathway. The interaction among the PKC, protein kinase A (PKA) and PKG pathways may further contribute to this blood-pressure-lowering effect [29,46,47].

#### 4.3.5. Potassium Channel Regulation

Research reports that CA can regulate multiple potassium channel subtypes, including voltage-gated K^+^ channel (K_V_7.2/7.3) and G-protein-activated inward rectifier K^+^ channels (GIRK4). This regulatory effect helps stabilize membrane potential, promote hyperpolarization of endothelial cells, and thereby facilitate the relaxation of vascular smooth muscle [53,54].

### 4.4. Reduction in Hypertension-Related Risk Factors

In addition to its direct vascular effects, CA can also improve systemic metabolic abnormalities that may lead to hypertension. It improves insulin sensitivity and lipid metabolism by activating AMPK and inhibiting sterol regulatory element-binding proteins (SREBPs), thereby reducing liver lipid accumulation and inflammation [26,27]. In metabolic syndrome and atherosclerosis models, CA can enhance antioxidant enzyme activity (superoxide dismutase, catalase), lower serum glucose and malondialdehyde levels, and downregulate NF-κB-mediated monocyte chemoattractant protein-1 expression [37,44]. These combined effects can alleviate endothelial stress and the atherosclerotic process, indirectly promoting blood pressure stability.

Overall, existing preclinical evidence suggests that the effects of corosolic acid are more model-dependent dose–response relationships than a uniform, fixed dose–response pattern. Evidence directly supporting its blood-pressure-lowering effect primarily comes from SHR/NDmcr-cp rats receiving dietary corosolic acid intervention; in contrast, mouse models and cardiac remodeling models mainly demonstrate its effects on metabolic improvement, anti-inflammation, anti-atherosclerosis, or cardioprotection. Therefore, current evidence supports the biological activity of corosolic acid in various cardiovascular and metabolic disease models, but the optimal dose for its use as an antihypertensive drug remains undetermined (Table 1).

### 4.5. Emerging and Unresolved Mechanisms

The latest evidence suggests that CA may stimulate mitochondrial autophagy through the prohibitin-2 (PHB2)/PTEN-induced kinase-1 (PINK1)/Parkin pathway, thereby alleviating oxidative damage in the cardiac ischemia model. However, it is unclear whether this mechanism directly participates in systemic blood pressure regulation [55]. Similarly, the potential effects of CA on calcium channel activity, autonomic nerve regulation, and isoform-specific regulation of protein kinases and nitric oxide synthases also warrants further investigation.

## 5. Additional Cardioprotective Effects

In addition to its direct blood-pressure-lowering mechanism, CA also has extensive cardioprotective effects, enhancing its potential in the treatment of cardiovascular diseases. Multiple in vivo studies have confirmed that CA, through mechanisms such as antioxidation, anti-fibrosis, and regulation of autophagy, can alleviate myocardial damage, limit pathological cardiac remodeling, and maintain ventricular function.

### 5.1. Protection Against Myocardial Infarction and Fibrosis

In the murine myocardial infarction model, treatment with CA significantly increased survival rate, reduced infarct size, and improved left ventricular systolic function. Histological analysis revealed decreased collagen deposition and reduced expression of fibrosis-related markers (such as transforming growth factor-β and α-smooth-muscle actin). These effects are attributed to the inhibition of oxidative stress by CA and the suppression of pro-inflammatory signals through the NF-κB and JAK/STAT pathways. Activation of AMPK and upregulation of heme oxygenase-1 also further enhanced cell protection and alleviated post-infarction remodeling [25,41].

### 5.2. Prevention of Cardiac Hypertrophy

The study also demonstrated that CA can counteract cardiac hypertrophy induced by excessive stress. In models of hypertension and aortic stenosis, CA can reduce the volume of myocardial cells, limit myocardial fibrosis, and normalize the expression of hypertrophy-related genes (such as atrial natriuretic peptide (ANP) and brain natriuretic peptide (BNP)). Mechanistically, these benefits are related to the regulation of autophagy and the control of the AMPK/mTOR signaling cascade, which maintain cellular energy balance and prevent inappropriate cardiac growth in response [24,25].

### 5.3. Mitochondrial and Metabolic Protection

Emerging evidence suggests that CA supports mitochondrial homeostasis by activating the mitochondrial autophagy pathway mediated by PHB2/PINK1/Parkin. By eliminating damaged mitochondria, CA limits excessive reactive oxygen species production and maintains the ATP synthesis efficiency in cardiac cells. These mitochondrial effects complement its role in systemic glycolipid metabolism, thereby reducing the metabolic burden on cardiac tissues [55].

### 5.4. Integrative Cardiovascular Benefits

Overall, the cardioprotective effects of CA, including reduction in oxidative and inflammatory stress, modulation of hypertrophic remodeling, and maintenance of mitochondrial function, may support vascular and myocardial health. These effects suggest that CA could be a versatile phytochemical candidate for addressing both the hemodynamic and metabolic aspects of hypertensive cardiovascular disease, although this interpretation remains primarily preclinical.

## 6. Examination of Study Constraints and Future Directions

Although evidence has accumulated to support the BP-lowering and cardioprotective actions of CA, current studies remain largely preclinical and require further investigation. Most research has been performed using cell culture models or animals, with a lack of direct evidence from human studies linking the mechanisms at the cell level and response to treatment. Therefore, several key limitations require further investigation.

### 6.1. Absence of Conclusive Data Regarding Calcium Channel Regulation

Whether CA modulates voltage-gated, receptor-operated, or store-operated calcium channels in vascular smooth muscular cells or endothelial cells remains to be determined as no studies have addressed this issue so far. In fact, these types of ion channels are critical for the excitation–contraction coupling and modulation of vasoconstriction. Although there are a few circumstantial data indicating that CA may modulate the intracellular Ca level by means of cyclic adenosine monophosphate (cAMP)/PKA pathway, as well as K^+^ channels, further electrophysiological studies will be needed to establish exactly how it modulates Ca^2+^ homeostasis.

### 6.2. Limited Knowledge Regarding Autonomic Nervous System Mechanisms

The development of hypertension has been related to the disturbed balance between sympathetic and parasympathetic activities. However, CA’s effect on the sensitivity of autonomic control or baroreceptors has not been studied in the previous literature. Given that several triterpenoid molecules have been reported to modulate neurotransmission in both the central and peripheral nervous systems, future studies need to address whether CA is able to modulate autonomic balance and heart rate variability in animal models of hypertension.

### 6.3. Unaddressed Selectivity Issues Regarding NOS and PKC Isoforms

While we know that CA enhances NO synthesis and inhibits PKC, it remains unclear if these effects occur at specific isoforms of either enzyme, considering that the three major NOS isoforms—eNOS, inducible NOS (iNOS) and neuronal NOS (nNOS)—exert distinctive, sometimes opposing actions on vascular function. Similarly, different PKC isoforms have differential roles for controlling vascular contraction, barrier function or inflammation. Using selective inhibitors, stimulators, or genetic knockdowns might reveal CA’s molecular target preferences and allow for designing more specifically acting therapeutic derivatives.

### 6.4. Limited Clinical and Pharmacokinetic Data

To date, no randomized controlled trials have evaluated the efficacy, tolerability, or dose-finding of CA for hypertension. Data on its pharmacokinetic processes, including absorption, bioavailability, metabolism, tissue distribution, and elimination, remain insufficiently characterized. These limitations prevent current preclinical findings from being directly translated into clinical recommendations. Standardization of CA formulations, dosage strategies, routes of administration, and dosage regimens is needed before considering its therapeutic use. Further research is also required to assess potential drug–drug interactions or drug–nutrient interactions, especially when CA is used in combination with antihypertensive or hypoglycemic agents.

From a translational research perspective, the potential interaction risks of CA should be considered in two clinically relevant scenarios. First, since existing studies have reported that CA can improve glucose uptake, insulin sensitivity, and AMPK/PPAR-γ/NF-κB-related metabolic regulation [13,16,26,30], it may theoretically increase the risk of excessive glucose lowering when used in combination with insulin, sulfonylureas, metformin, GLP-1 receptor agonists, SGLT2 inhibitors, or other hypoglycemic agents, especially in diabetic patients, those with reduced food intake, or those with impaired renal function. Secondly, since CA may promote vasodilation through NO/cGMP, H_2_S/K_ATP_, and PKC-related pathways and has shown antihypertensive effects in preclinical models [18,21,23,29], it is theoretically possible that co-administration with ACE inhibitors, angiotensin receptor blockers, calcium channel blockers, diuretics, beta-blockers, nitrates, or other vasodilators could increase the risk of hypotension, dizziness, altered renal perfusion, or electrolyte disturbances. Currently, these risks remain theoretical speculations because there are no dedicated clinical interaction studies, pharmacovigilance studies, CYP450/UGT enzyme studies, transporter studies, or combination therapy studies assessing the interaction of CA with antihypertensive or hypoglycemic agents. Therefore, before considering CA-containing formulations for adjunctive clinical treatment, future translational studies should include medication history screening, blood glucose and blood pressure monitoring, pharmacokinetic/pharmacodynamic interaction assessments, and safety endpoints for hypoglycemia, symptomatic hypotension, renal function, and electrolyte disturbances.

Compared to traditional antihypertensive drugs, CA should currently be considered as a component with a different mechanism of action rather than a direct replacement for drugs. ACE inhibitors and angiotensin receptor blockers mainly work by reducing Ang II production or blocking AT1R-mediated vasoconstriction signals, while calcium channel blockers mainly reduce vascular smooth muscle contraction by inhibiting L-type calcium channel-mediated calcium influx [4,5,6]. In contrast, existing evidence suggests that CA may act through a broader but clinically undervalidated network, including indirectly modulating RAS-related downstream signaling [25,40], enhancing NO/cGMP and H_2_S/K_ATP_-mediated vasodilation [18,23], inhibiting PKC-related vasoconstriction signals [29], and alleviating oxidative stress, inflammation, and metabolic dysfunction through AMPK/PPAR-γ/NF-κB-related pathways [13,16,21,26,30]. This multi-target characteristic provides a theoretical basis for the use of CA, as adjunctive therapy in hypertensive patients with endothelial dysfunction, insulin resistance, obesity, or chronic vascular inflammation. However, unlike ACE inhibitors, angiotensin receptor blockers, or calcium channel blockers, CA currently lacks randomized clinical trials, standardized dosages, and validated pharmacokinetic/pharmacodynamic interaction data. Therefore, their potential combination therapy value should be cautiously understood as a preclinical hypothesis. Future research should compare CA with standard antihypertensive drugs and assess whether combination therapy can provide additional vascular or metabolic benefits without increasing the risk of hypoglycemia, hypotension, renal dysfunction, or electrolyte-related adverse events.

### 6.5. Apoptosis, Autophagy, and Endothelial Homeostasis

While CA has been studied in apoptosis and autophagy in tumors, its involvement in vascular homeostasis remains unclear. Further studies are required to elucidate if this process modulates vascular remodeling, endothelial cell regeneration, or antioxidant response in hypertension.

### 6.6. Investigation of Endothelial Function and Upstream Targets

Further studies using endothelial and vascular smooth muscle cells cultures need to be performed to evaluate the effect of CA on endothelin-1 secretion, ROS production, and other vasoactive mediators. Further target identification strategies should be employed to determine the main molecular targets responsible for initiating downstream effects of CA, such as affinity-based experiments, molecular interaction studies, and high-throughput omics methods. These studies may contribute to a deeper understanding of the compound’s mechanism of action.

### 6.7. Incorporation into Clinical Research Paradigms

Extrapolation of findings from animals to humans requires integration of metabolic profiling, biomarker identification, and mechanistic analyses. This holistic approach helps determine whether the cardiovascular regulatory effects observed in preclinical models can translate into vascular regulation and cardiovascular metabolic benefits in humans.

## 7. Conclusions

In recent years, CA has been reported as an emerging multifunctional phytochemical with potential antihypertensive properties [11,21,22,44]. It acts through several and sometimes overlapping pathways such as RAS-associated signaling [19,20,25,40], antioxidative and anti-inflammatory effects [21,23,26,27,39,42,44], enhancement of vasorelaxation signals, including NO and H_2_S [18,23,45], and modulation of key enzyme systems such as AMPK, NF-κB, JAK/STAT, and PKC [16,24,25,26,27,28,29,43]. These effects are likely to be additive towards vasorelaxation, endothelial function improvement and reduction in the overall cardiovascular risk profile associated with hypertension [21,23,39,43,44].

However, the precise molecular targets of CA remain largely undefined, especially concerning its isoform-specific effects on NOS or PKC [18,29,45,46,47]. Consequently, well-designed pharmacokinetic, toxicological, and clinical studies are essential to validate its efficacy, define safe therapeutic dosing, and elucidate potential drug–nutrient interactions [11,44].

In conclusion, based on its multi-target mechanism of action, CA may be a promising preclinical adjuvant candidate for hypertension-related vascular and cardiometabolic dysfunction [16,18,21,23,26,27,39,43]. However, this conclusion should be interpreted with caution, as current evidence primarily comes from animal experiments and in vitro studies [21,23,24,25,26,27,39,41,43,55]. In the absence of human clinical trials and sufficient pharmacokinetic data, CA cannot currently be considered an evidence-based adjuvant therapy for hypertension [11,44]. Its translation into clinical practice still requires rigorous pharmacokinetic, toxicological, dosage exploration, and randomized clinical trials to link laboratory mechanistic evidence with clinically meaningful blood pressure control and cardiovascular protective effects [11,44].

## Figures and Tables

**Table 1:** Model-dependent dosing regimens and dose–response patterns of corosolic acid in preclinical cardiovascular and metabolic studies.

Study and Animal Model	CA Dosing Regimen and Treatment Duration	Main Cardiovascular or Metabolic Outcomes	Interpretation of Dose–Response Evidence
Yamaguchi et al. [21], 2006; SHR/NDmcr-cp rats with metabolic syndrome-related hypertension.	0.072% CA in a high-fat diet for 14 weeks.	Blood pressure, serum-free fatty acids, oxidative stress and inflammatory markers; CA reduced elevated blood pressure by about 10% after 8 weeks and decreased oxidative/inflammatory markers.	Most direct antihypertensive evidence, but only one dietary dose was tested; therefore, efficacy is supported without a graded dose–response curve.
Li et al. [23], 2022; high-fat/high-sugar-diet-induced metabolic syndrome erectile dysfunction rats.	20 mg/kg/day by oral gavage for 4 weeks.	ICP/MAP ratio, ROS, cGMP, eNOS and gp91phox; CA improved erectile function, reduced ROS/gp91phox and increased cGMP/eNOS.	Supports endothelial NO-related vascular protection in metabolic syndrome; indirectly relevant to hypertension, not a direct BP dose-ranging study.
Yang et al. [48], 2016; high-fat-diet-fed C57BL/6 mice with insulin resistance and adipose inflammation.	10 and 20 mg/kg by oral administration in a low-/high-dose comparison.	Body weight, adipocyte size, glucose intolerance, hyperlipidemia, adipose inflammation and AMPK signaling; stronger metabolic and anti-inflammatory effects were generally observed at 20 mg/kg.	Clearest two-dose comparison; supports metabolic risk factor improvement rather than direct blood pressure lowering.
Chen et al. [37], 2012; ApoE-deficient mice fed a Western-type/high-fat diet.	10 mg/kg/day by chow-based administration for 12 weeks.	Atherosclerotic lesion area, MCP-1, CCR2 and NF-kappaB signaling; CA reduced lesion area and inflammatory signaling.	Supports vascular anti-inflammatory and anti-atherosclerotic action; only one dose was tested.
Kawade et al. [43], 2023; monocrotaline-induced pulmonary hypertension rats and PASMCs from IPAH patients.	1 mg/kg/day in rats; 0.1–30 uM in PASMCs for the in vitro concentration response assay.	RV systolic pressure, pulmonary vascular remodeling, right ventricular hypertrophy, PASMC proliferation/migration and STAT3; CA attenuated PAH remodeling and inhibited PASMC proliferation concentration-dependently.	Strong vascular remodeling evidence; in vitro concentration–response assay was clear, whereas in vivo testing used a single low dose.
Wang et al. [25], 2019; aortic banding-induced cardiac hypertrophy in C57BL/6J mice.	10 and 20 mg/kg/day by daily gavage/irrigation for 6 weeks total.	Cardiac hypertrophy, fibrosis, dysfunction, LC3-II and p-AMPK; CA attenuated hypertrophy/fibrosis and activated AMPK-dependent autophagy.	Two-dose cardioprotective evidence relevant to hypertensive cardiac remodeling, but not a direct antihypertensive model.
Wang et al. [41], 2020; myocardial infarction model in C57BL/6J mice.	10 and 20 mg/kg/day; 14 days pretreatment plus 4 weeks after MI.	Survival, ventricular function, cardiac fibrosis, oxidative stress, inflammation, apoptosis and TGF-beta/Smad; CA improved function and reduced remodeling.	Supports cardiovascular protection at 10–20 mg/kg/day; informs cardiac injury protection rather than BP lowering.
Alkholifi et al. [16], 2023; STZ-induced diabetic rats with isoproterenol-induced myocardial injury.	50 mg/kg/day by oral route for 28 days.	SAP, DAP, MAP, heart rate, CK-MB, LDH, oxidative stress, inflammatory cytokines and PPAR-gamma; CA improved hemodynamic and myocardial injury indices.	Single relatively high-dose rat study; useful for diabetic cardioprotection and hemodynamic impairment, but not hypertension-specific dose ranging.
Yamada et al. [49], 2008; high-fat-diet-fed KK-Ay genetically obese mice.	0.023% CA in a high-fat diet for 9 weeks.	Body weight, fat mass, glucose, insulin, triglycerides, adiponectin and PPAR signaling; CA reduced body weight, fat mass and metabolic abnormalities.	Supports dietary CA effects on obesity and insulin resistance; indirectly relevant to hypertension through metabolic risk reduction.

Abbreviations: BP, blood pressure; CA, corosolic acid; HFD, high-fat diet; ICP/MAP, intracavernosal pressure/mean arterial pressure; IPAH, idiopathic pulmonary arterial hypertension; PASMCs, pulmonary arterial smooth muscle cells; PAH, pulmonary arterial hypertension; ROS, reactive oxygen species; RV, right ventricular; SHR, spontaneously hypertensive rat; STZ, streptozotocin.

## Data Availability

Data available in a publicly accessible repository.

## Abbreviations

AMPKα	adenosine monophosphate-activated protein kinase α
AMPK	AMP-activated protein kinase
ANP	atrial natriuretic peptide
BNP	brain natriuretic peptide
BP	blood pressure
CA	corosolic acid
cAMP	cyclic adenosine monophosphate
cGMP	cyclic guanosine monophosphate
COX	Cyclooxygenase
eNOS	endothelial NOS
ERK 1/2	extracellular signal-regulated kinase 1/2
FAK	focal adhesion kinase
GIRK4	G-protein-activated inward rectifier K^+^ channels
HO-1	heme oxygenase-1
H_2_S	hydrogen sulfide
IKKβ	inhibitor of nuclear factor kappa-B kinase
iNOS	inducible NOS
JAK/STAT	Janus kinase-signal transducer and activator of transcription
K_ATP_	ATP-sensitive potassium channel
K_V_	the voltage-gated K^+^ channel
NF-κB	nuclear factor kappa-B
nNOS	neuronal NOS
NO	nitric oxide
NOS	nitric oxide synthase
Nrf2	nuclear factor erythroid 2-related factor 2
PHB2	prohibitin-2
PINK1	PTEN-induced kinase-1
PKA	protein kinase A
PKC	protein kinase C
PKG	protein kinase G
PPAR-γ	peroxisome proliferator-activated receptor γ
RAS	renin–angiotensin-system
ROS	reactive oxygen species
sGC	soluble guanylyl cyclase
SREBPs	sterol regulatory element-binding proteins
